# The Use of Ovarian Fluid as Natural Fertilization Medium for Cryopreserved Semen in Mediterranean Brown Trout: The Effects on Sperm Swimming Performance

**DOI:** 10.3390/vetsci10030219

**Published:** 2023-03-13

**Authors:** Giusy Rusco, Michele Di Iorio, Stefano Esposito, Emanuele Antenucci, Alessandra Roncarati, Nicolaia Iaffaldano

**Affiliations:** 1 Department of Agricultural, Environmental and Food Sciences, University of Molise, 86100 Campobasso, Italy; 2 Mediterranean Trout Research Group “MTRG”, 42032 Ventasso (RE), Italy; 3 School of Biosciences and Veterinary Medicine, University of Camerino, 62024 Matelica, Italy

**Keywords:** fertilization medium, frozen semen, artificial reproductive microenvironment, conservation project

## Abstract

**Simple Summary:**

In the context of the “Life—Nat.Sal.Mo” project, obtaining an effective semen cryopreservation protocol was an important milestone that allowed the implementation of the first European cryobank of native Mediterranean brown trout (*S. cettii*) inhabiting Molise rivers (Italy). The main use of our semen cryobank is represented by its practical application in artificial reproduction practices aimed at maximizing the genetic variability of the offspring and reducing the genetic introgression in the native trout populations in the project area. Thus, the choice of the most suitable activation/fertilization medium represents the last key step in the development of artificial reproduction protocols using cryopreserved semen. Therefore, the aim of the present study was to investigate the effect of ovarian fluid as a natural activation media of spermatozoa on the post-thaw sperm swimming performance of Mediterranean trout, comparing it with D-532 and a mixed solution of 50% D-532 and 50% ovarian fluid. Our results suggest that the presence of ovarian fluid alone or in combination with D-532 in the artificial microenvironment of reproduction represents a key factor to increase the success of fertilization when the frozen semen of Mediterranean brown trout is used.

**Abstract:**

D-532 fertilization solution is generally used to replace the water or ovarian fluid during artificial reproductive practices in salmonids due to its ability to boost sperm motility and increase fertilization rates compared with natural activation media. However, the maintenance of ovarian fluid in a reproductive microenvironment gives it the advantage of protecting the eggs from potential harmful factors from the external environment and simplifying the field operations related to its removal when D-532 is used alone. In light of this, the aim of the present study was to investigate in vitro, for the first time, the effect of ovarian fluid (OF 100%) on post-thaw sperm swimming performance of Mediterranean trout, comparing it with D-532 and a mixed solution of 50% D-532 and 50% ovarian fluid (OF 50%). The percentage of motile spermatozoa and movement duration was significantly increased in OF 100% and OF 50% compared with D-532. Sperm velocity was higher in D-532, but significant differences were recorded only with OF 100%. In conclusion, these results suggest that the presence of ovarian fluid alone or in combination with D-532 in an artificial microenvironment of reproduction represents a key factor in potentially increasing fertilization success when the frozen semen of Mediterranean brown trout is used.

## 1. Introduction

In the context of conservation projects, cryobanks are a valuable tool to preserve the genetic resources of a wide range of species, providing a crucial opportunity to support the artificial reproduction practices aimed to increase the genetic variability of offspring [1,2,3]. Sperm cells are the main type of cells used for cryobanking purposes in aquatic species. In fact, thanks to their small size and relatively high resistance to chilling, sperm cryopreservation is a more feasible method compared with the ones performed on other cell types [3]. The obtainment of an effective semen cryopreservation protocol was an important milestone in our project (LIFE Nat.Sal.Mo.), that allowed us to implement the first European cryobank of the native Mediterranean brown trout (*S. cettii*) inhabiting Molise rivers [4,5]. The main use of our semen cryobank is represented by its practical application in artificial reproduction practices. Indeed, frozen semen doses of native males are used in cross-fertilization schemes within artificial reproduction to maximize the genetic variability of offspring and reduce the genetic introgression in the native trout populations within the project area. An important prerequisite for successfully fertilizing eggs is the sperm swimming performance. In this regard, it is known that an appropriate reproductive microenvironment is crucial to promote a good activation of sperm motility and their simultaneous encounter with eggs; this result is particularly important in external fertilizer fish [6,7]. For this reason, the selection of the most suitable activation/fertilization medium represents a crucial step in the artificial reproduction protocols, especially when cryopreserved semen is adopted. Generally, artificial fertilization media are developed in order to boost sperm motility and increase fertilization rates compared with natural media, such as water or ovarian fluid [8,9,10,11,12,13,14,15]. In particular, among more effective artificial fertilization media, D-532 saline solution was used to replace ovarian fluid during the artificial fertilization practices in salmonids in order to avoid the variability problems related to the composition of ovarian fluids and their possible contamination with yolk from broken eggs [10]. In this regard, in our previous studies aimed at fine-tuning the semen cryopreservation protocols for native Mediterranean brown trout [4,16], we used D-532 solution for the in vivo fertilization trials in order to reduce the variability of the results due to the differences in the quality of the ovarian fluid from individual females as much as possible, making the microenvironment of fertilization more homogeneous. Nevertheless, in subsequent fertilization practices with frozen sperm performed within the project Nat.Sal.Mo. on the river bank, ovarian fluid alone as a natural activation medium of sperm motility was used for two main reasons: (1) to maintain the artificial microenvironment of reproduction as similar as possible to that of natural spawning, protecting the eggs from potential harmful factors from the external environment; (2) to reduce the time required for the complete removal of ovarian fluid when D-532 is used alone. Surprisingly, we noted that the use of ovarian fluid alone registered generally higher fertilization rates [5] than those we found during the in vivo trials conducted in the presence of D-532 [4,16].

The ability of ovarian fluid to significantly affect the swimming performance of fresh sperm and consequently influence the outcome of fertilization in terms of fertilized oocyte rate has been shown in several fish species [14,17,18,19,20,21,22,23,24,25,26,27,28,29]. However, to the best of our knowledge, there are no studies that have tested the effect of ovarian fluid on the motility parameters of frozen semen.

In light of these considerations and given the optimal fertilization rates obtained within our project using ovarian fluid alone, we investigated, for the first time, the in vitro effect of ovarian fluid on the post-thaw sperm of Mediterranean trout’s swimming performance and compared it with D-532 fertilization solution. Moreover, a mixed solution that included 50% D-532 and 50% ovarian fluid was also tested to evaluate the potential use of this combined medium in order to avoid the elimination of ovarian fluid, simplifying the field operations.

## 2. Materials and Methods

### 2.1. Animals and Collection of Samples 

During the breeding season (January–February 2021), 17 breeders (7 males and 10 females) of native Mediterranean brown trout were captured in the Biferno river by electro-fishing. The males belonged to the 2+ and 3+ classes, with an average total length of 30.2 ± 3.9 cm; the females were 3+ and 4+ years old and characterized by an average total length of 44.1 ± 5.2 cm.

Semen samples (N = 7) were collected by gentle abdominal massaging, drying the abdomen and urogenital papilla with special care before stripping in order to avoid contamination of semen with urine, mucus and blood cells. 

Eggs with their own ovarian fluids (OFs) were collected by the same method described above for males, and ovarian fluids were separated from egg batches of each female (N = 10) directly with a syringe after egg decantation.

After sample collection, both males and females were immediately released into the water course.

The sperm and OF samples were transferred from the river to the laboratory in a cooler that contained ice and processed within 4 h of the collection. Semen samples were subjected to the cryopreservation procedure optimized in our previous work [4]. Briefly, each semen aliquot was diluted with a freezing extender to reach 0.15 M of glucose, 7.5% of methanol and a sperm concentration of 3.0 × 10^9^ sperm/mL. The diluted semen was charged into 0.25 mL plastic straws and equilibrated for 15 min on ice (at the height of 3 cm). Lastly, the straws were cryopreserved by exposure to liquid nitrogen (LN_2_) vapor at 3 cm above the LN_2_ level for 5 min and plunged into LN_2_. 

The OFs were subjected to pH measurement using a BasiC 20 pH meter (CRISON instruments, Barcelona, Spain) and subsequently stored at −20 °C until osmolality measurement by an OSMOMAT 3000 basic (GONOTEC, Reuchlinstr, Berlin, Germany), according to the manufacturer’s protocol. OF samples were frozen because of the impossibility to collect all of the sperm and ovarian fluid samples on the same day [30].

### 2.2. Experimental Design

Sperm motility analyses were performed using a Computer-Assisted Sperm Analysis (CASA) system coupled to a phase contrast microscope (Nikon model Ci-L) employing the Sperm Class Analyzer (SCA) software (VET Edition, Barcelona, Spain), that carried out two replicate sperm activations for each treatment combination.

The experiment was conducted over 11 days. On the 1st day, the semen samples (N = 7) were analyzed after thawing at 40 °C for 5 s using two different activation media: 1% NaHCO_3_ as a control solution (C) to test cryopreserved semen quality and D-532 as a benchmark artificial fertilization solution (20 mM Tris, 30 mM glycine, 125 mM NaCl, pH 9.0), at a dilution ratio of 1:30 (*v*:*v*). On each of the remaining 10 days, one microtube of OF from a single female (N = 10) was thawed at 4 °C for 2 h and split into 2 aliquots: one was used as OF 100%, and the other diluted at 50% with the D-532 fertilization solution (OF 50%). Subsequently, the thawed semen from each male was activated in OF 100% and OF 50%, using the same dilution ratio reported above. After rapid mixing, 3 µL of diluted semen was immediately loaded onto a 20-micron Leja slide (Leja Standard Count, Nieuw Vennep, The Netherlands) and evaluated by the computer-assisted sperm analysis system (CASA). Sperm motility was analyzed 10 s post-activation, acquiring videos at 25 fps rate. The following sperm traits were evaluated: motile spermatozoa (MOT, %), curvilinear velocity (VCL, µm/s), straight-line velocity (VSL, µm/s), average path velocity (VAP, µm/s), linearity (LIN, %), straightness (STR, %), beat cross frequency (BCF, Hz) and amplitude of lateral displacement of the spermatozoon head (ALH, µm). The duration of sperm movement (DSM) was measured using a chronometer from sperm activation to movement cessation of spermatozoa in the field of view. The loading of chambers and the recording of motility parameters were both carried out by the same operator, taking care to standardize the operation timing from ice to observation.

Sperm motility is nowadays considered the best sperm quality biomarker in fish as they are strongly correlated with fertilization ability. In this regard, the CASA system is the most objective and comprehensive quantification method currently available to assess several sperm motility parameters in various species, including fish [31].

### 2.3. Statistical Analysis

All statistical analyses were performed using the statistical software R (Version 4.2.0), at significance levels of *p* ≤ 0.05. The replicates were treated as repeated measures, using its mean for statistical analysis. MOT (%) measurements were subjected to prior *arcsine* transformation. The correlations among sperm motility parameters were analyzed in order to identify closely related parameters and reduce the number of parameters to be addressed and discussed. 

Normality and homoscedasticity were tested by visual inspection of the residuals’ graphs, Shapiro–Wilk test and Levene’s test (*rstatix* and *car* R packages). The differences among treatments for all tested sperm motility parameters were analyzed using Mixed Model ANOVAs with the fertilization medium as a fixed factor and the ID male as a random factor, followed by Tukey’s post-hoc test for multiple comparisons between treatments (*lme4*, *lmerTest* and *multcomp* R packages). Furthermore, Mixed Model ANOVAs were conducted, isolating the OF groups to test the differences between OF 100% and OF 50%, adding the random effect of females, males and their interaction. The AIC test was used to compare different models and determine which one was the best fit for the data (*lmerTest* R package).

To evaluate the association between the quality of cryopreserved semen assessed through the activation in C and its performance in the fertilization media, the correlations between sperm motility parameters registered in OFs (100% and 50%) and in the control activation solution (C) were tested using the Pearson parametric test (*Hmisc* and *PerformanceAnalytics* R packages).

All graphics were generated using the *ggplot2* package.

## 3. Results

The average (±SD) osmolality and pH of the ovarian fluids (N = 10) were 234.7 ± 74.77 mOsm/kg and 8.63 ± 0.17, respectively. The three velocities (VCL, VAP and VSL) and two linearity parameters (STR and LIN) resulted as strictly associated. Therefore, we decided to reduce the set of showed and discussed parameters to six (MOT, VCL, LIN, ALH, BCF, DSM). All sperm motility parameters and the correlation matrix are reported in Supplementary Material (Table S1 and Figure S1).

### 3.1. Comparison of the Post-Thaw Sperm Motility Parameters among the Different Activation Media 

**Figure 1:**
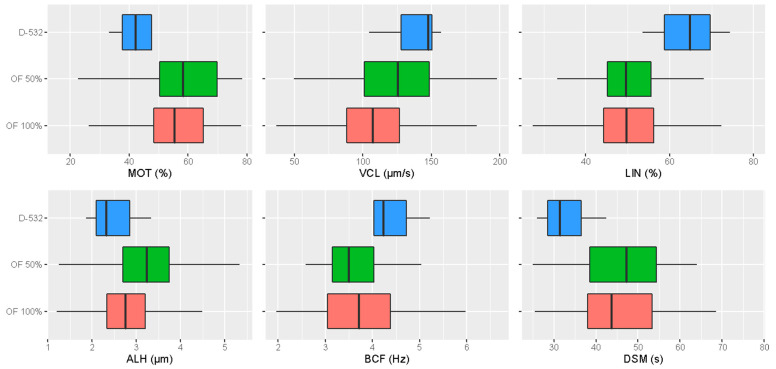
Boxplots displaying the “within” and “between” treatments variability concerning the main sperm motility parameters (outliers are not shown).

The significant differences among the post-thaw sperm motility parameters activated in OF 100%, OF 50% or D-532 are shown in Table 1. The activation solutions that included the ovarian fluids (OF 100% and OF 50%) induced a significant increase in total sperm motility (MOT) and its duration (DSM) compared with D-532. However, a significant increase in VCL was observed using D-532 and OF 50% with respect to OF 100%. Linearity (LIN) was higher when spermatozoa were activated in D-532. Furthermore, significantly higher values of lateral displacement amplitude of the spermatozoon head (ALH) were found for cryopreserved semen activated in OF 50%. No differences among different treatments for BCF were found. In Figure 1, the variability range of motility parameters is shown. A wider variability is observed when ovarian fluid is used.

### 3.2. Differences between OF 100% and OF 50%: The Effect of Males, Females and Male–Female Interaction

The results for the best-fitting mixed models are reported in Table 2. For MOT, VCL and ALH, a significant difference was found between OF 100% and OF 50%. In particular, MOT and ALH were significantly higher in OF 50%, with an interaction effect between males and females. VCL was also higher in OF 50%, with the best model represented by the only random effect of inter-male variability, according to pairwise comparison in Section 3.1 (Table 2). No differences were observed for LIN, BCF and DSM between OF 100% and OF 50%.

### 3.3. Correlation among the Post-Thaw Sperm Motility Parameters Activated in Ovarian Fluid and Control Activation Solution

**Figure 2:**
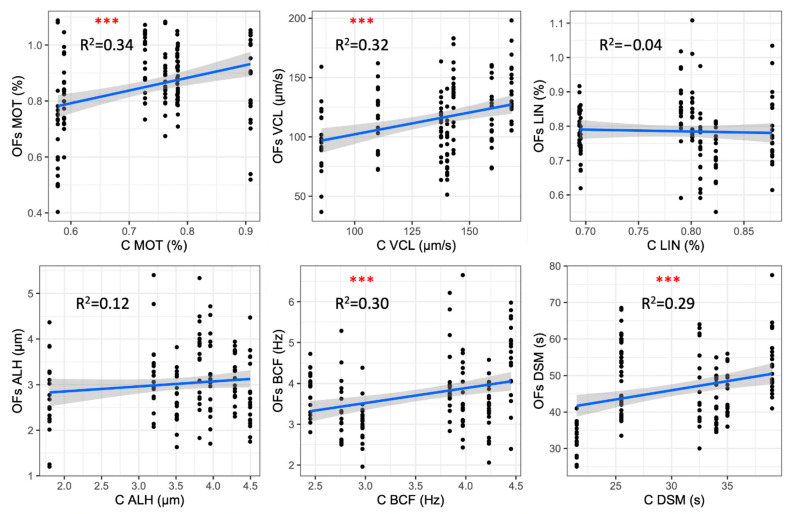
Correlation among the post-thaw sperm motility parameters using ovarian fluid and control activation solution. Significance levels (in red) and coefficients of determination (R^2^) are reported. ***: *p* < 0.001.

The MOT, VCL, BCF and DSM values recorded using OF (OF 50% and OF 100%) were positively correlated with those obtained in the control activation solution (C). No relationship was identified for LIN and ALH (Figure 2). 

## 4. Discussion

For the first time, the effect of ovarian fluid on the post-thaw sperm motility parameters of Mediterranean brown trout *S. cettii*^1^ was tested, and it was compared both with an artificial fertilization medium (D-532) and ovarian fluid supplemented with 50% D-532.

Our results showed that the activation solutions with ovarian fluid (OF 100% and OF 50%) significantly prolonged the duration of movement (longevity) compared with D-532 alone, confirming the on-field observation of the fertilization success obtained during the LIFE Nat.Sal.Mo. project [5]. 

It is possible that some chemical constituents of ovarian fluid influence ATP metabolism, such that the duration of energy production is increased [17]. Total motility was also higher in OF 100% and OF 50%; however, significant differences were found only between OF 50% and D-532. On the contrary, when D-532 was used, the sperm velocity parameters underwent an overall increase at the potential expense of longevity. It is, in fact, possible that a higher swimming velocity required higher energy consumption by spermatozoa, thus leading to a lower movement duration, as suggested by Cosson [32]. In addition, from these results, it is interesting to note the potential positive effect exercised by the combination of ovarian fluid and D-532 (OF 50%) on the post-thaw sperm motility parameters. The presence of D-532 solution in the OF, in fact, significantly increased the velocity parameters compared with OF alone; at the same time, the presence of OF in D-532 solution significantly improved both the total motility and the duration of movement in comparison to the D-532 buffer alone. The prolongation of the duration of sperm movement obtained by adding 50% of ovarian fluid to the D-532 buffer is consistent with findings found by Dietrich et al. [33] using fresh semen from rainbow trout (*Oncorhynchus mykiss*). The high standard deviation registered for some parameters is explained by differences between males (the random factor in our mixed model). Furthermore, we observed that the presence of ovarian fluid in the activation media increased the variability of results (Figure 1), which could be mainly explained by the inter-female variability and/or the male x female interaction effect (Supplementary Material: Figures S2–S4). The female effect is generally attributed to the intrinsic differences in the composition of ovarian fluid from individual females, such as affecting sperm motility characteristics differently [34,35,36]. At the same time, the male effect is due to the different intrinsic sperm qualities existing among individual males. In this regard, some authors [22,26] suggested that some males produce sperm with superior motility characteristics despite the variability in the motility-modulating activity of particular ovarian fluid. 

We found positive correlations for many of the sperm motility parameters (MOT, VCL, BCF, and DSM; Figure 2) among the solutions containing ovarian fluid (OF 100% and OF 50%) and the control solution, suggesting that sperm of good quality, once activated, moves with similar parameters regardless of the activation media. The correlations for MOT, VCL, BCF and DSM are all characterized by *p* < 0.001 and R^2^ around 0.3. For such kinetic parameters, the combination of high significance levels and low R^2^ suggests that the performances in OFs were significatively correlated with the control values (activation in C medium) registered for each male, but they do not explain a great part of the variability that most likely arises from females and/or male–female effects. Thus, these results show that the activation in the control solution is a good proxy for the swimming performance of post-thawed cryopreserved sperm in the fertilization environment.

However, the limited or negative effect of ovarian fluid on spermatozoa performance is often associated with the presence of contaminants, such as vitellus from broken eggs, blood, water, feces, etc. [12,34,37,38,39,40]. In this regard, in order to counteract the negative effects of contamination, some authors recommend to discard ovarian fluid from salmonid eggs before fertilization and to use only isotonic diluents of pH 8.4–9.0 to enhance sperm motility [8,10,11,12,13,14]. Nevertheless, the composition of ovarian fluid that includes ions, proteins, amino acids and sugar is ideal to support and protect the eggs and, at the same time, extend the fertilization period both in the natural and artificial fertilization environment [41,42,43,44,45]. In light of this, we considered that leaving the ovarian fluid as natural sperm activation media during artificial reproduction practices carried out on the riverbank within our project was good practice. OF, as well as maintaining the advantage to maintain the artificial microenvironment of reproduction as similar as possible to that of natural spawning, allowed us to facilitate the management of field operations, avoiding the preparation, transport and the use of fertilization solutions. The use of D-532 alone would require the complete removal of ovarian fluid through a sieve; this time-consuming operation, in an external uncontrolled environment, exposes the eggs to possible freezing damage when temperatures are below zero; therefore, giving up the natural protection provided by the ovarian fluid.

Based on our results, the addition of a portion of D-532 to the eggs in the presence of their ovarian fluid could be another possible solution in order to facilitate field operations. However, we believe that this step is not indispensable because, although it is known that velocity is positively correlated with fertilization rates [46], it may not be a key parameter for successful fertilization when cross-fertilization schemes are adopted within artificial reproduction practices. From an application point of view, these fertilization schemes consist of splitting the eggs from each native female into equal aliquots so each of them can be fertilized with a frozen semen dose from different males. Therefore, a controlled reproductive microenvironment is created (in the absence of male competition) that already favors the gametes encounter. Moreover, since it is known that the cryopreservation process causes cell damage and compromises sperm motility and longevity [47], obtaining a greater number of post-thaw motile sperm/eggs that move for a longer time could potentially enhance fertilization success. To corroborate the above, higher fertilization rates, ranging from 64% to 81%, were achieved when only ovarian fluid was used [5] compared with those obtained in our previous papers (ranging from 53% to 65%) by also adding to eggs, the D-532 buffer [4,16].

^1^*Mediterranean brown trout is listed in Annex II of the Habitat Directive as Salmo macrostigma and currently reported as Salmo cettii in the Italian and European Red Lists and in the Member States’ conservation status assessments of the “habitats types and species of Community interest” (Art. 17). Recent studies suggest that Italian peninsular Mediterranean brown trout belong to a separate taxon named S. ghigii, limiting the name S. cettii to Sicilian trout* [48,49,50]. *Although we agree with the recent observations, we still use the name S. cettii in the current paper, because Mediterranean brown trout populations are still protected by the Habitat Directive and subsequent updates on its conservation status.*

## 5. Conclusions

The use of cryobanks supporting the conservation of endangered native species could solve or mitigate the known problems affecting the supportive breeding and the management of broodstocks, contributing to maintain the wild genetic biodiversity and avoiding domestication. To extend the use of these practices, the definition of operational cryopreservation and fertilization protocols is crucial. In this regard, this study suggests that the presence of ovarian fluid alone or in combination with D-532 in the artificial microenvironment of reproduction represents a key factor to increase the success of fertilization when frozen semen of Mediterranean brown trout is used. Notably, OF 100% is a natural, viable media for simplifying and expediting the field operations that occur on riverbank. 

In the future, it will be interesting to characterize the ovarian fluid biochemistry from native Mediterranean trout in order to investigate the components that can impact the in vitro and in vivo performance of fresh and frozen semen and to standardize fertilization protocols in controlled reproduction. Finally, the obtained results could be useful not only for the conservation of the native Mediterranean trout, but also to stimulate further research on the use of pure or mixed ovarian fluid as fertilization medium in other species of aquaculture interest.

## Figures and Tables

**Table 1:** Main sperm motility parameters (means ± SD) in D-532, OF 50% and OF 100% and pairwise comparison among treatments.

	Treatments		
Sperm Traits	D-532	OF 50%	OF 100%
MOT (%)	43.04 ± 13.59 ^b^	57.49 ± 14.00 ^a^	55.56 ± 13.83 ^ab^
VCL (µm/s)	138.01 ± 18.74 ^a^	122.29 ± 32.01 ^a^	107.72± 27.69 ^b^
LIN (%)	64.23 ± 7.79 ^a^	50.00 ± 8.55 ^b^	49.98 ± 9.90 ^b^
ALH (µm)	2.49 ± 0.57 ^b^	3.21 ± 0.80 ^a^	2.84 ± 0.70 ^b^
BCF (Hz)	4.28 ± 0.72	3.71 ± 0.84	3.72 ± 0.94
DSM (s)	32.86 ± 6.33 ^b^	46.39 ± 9.60 ^a^	45.98 ± 10.84 ^a^

^ab^ Different superscript letters within the same row indicate a significant difference (*p* < 0.05).

**Table 2:** Selection of the best-fitting model for Mixed Model ANOVAs isolating the OF groups, with the treatment (OF 100% vs. OF 50%) as a fixed factor and male, female or their interaction as random factors. Only the parameters with significative differences among treatments are shown.

Random Effects	MOT		VCL		ALH	
	*p*	AIC	*p*	AIC	*p*	AIC
Male	***	−154.41	***	1308.1	**	320.14
Female	*	−129.15		1346.2	**	320.5
Male–Female	***	−216.94	***	1329.2	***	309.83
Fixed effect						
Treatment (OF 100% vs. OF 50%)	*		***		***	

*p* < 0.05 *; *p* < 0.01 **; *p* < 0.001 ***. The best-fitting models (lowest AIC values) are marked in bold

## Data Availability

Not applicable.

## Supplementary Materials

The following supporting information can be downloaded at: https://www.mdpi.com/article/10.3390/vetsci10030219/s1, Table S1: All sperm motility parameters (means ± SD) in C, D-532, OF 50% and OF 100% and pairwise comparison among treatments; Figure S1: Correlation matrix among all the sperm motility traits; Figure S2: Barplots showing the additive male effect on total motility (MOT) and duration (DSM) grouping all treatments; Figure S3: The barplots show the additive female effect (mean of all male crosses) on total motility (MOT) and duration (DSM) grouping OF 50% and OF 100 %. Values registered in C and D.532 are shown; Figure S4: The barplot shows the interaction effect between male and female on total motility (MOT) grouped by male (each coloured bar) and female (each group of bars). Both females and male are sorted by their overall means.
