# Supplementary material for: The Use of Ovarian Fluid as Natural Fertilization Medium for Cryopreserved Semen in Mediterranean Brown Trout: The Effects on Sperm Swimming Performance
Source: Vet Sci. 2023 Mar 13;10(3):219. doi: 10.3390/vetsci10030219 (PMC10051816; doi:10.3390/vetsci10030219)
Supplement: Supplementary file 1 [file vetsci-10-00219-s001.zip › vetsci-2212927-supplementary.pdf]

Supplementary materials

Table S1. All sperm motility parameters (means ± SD) in C, D-532, OF 50% and OF 100% and pairwise comparison among treatments.

	**Treatments**			
**Sperm traits**	**C**	**D-532**	**OF 50%**	**OF 100%**
MOT (%)	44.88 ± 11.40	43.04 ± 13.59	57.49 ± 14.00	55.56 ± 13.83
VCL (μm/s)	134.91 ± 28.52	138.01 ± 18.74	122.29 ± 32.01	107.72± 27.69
VAP (μm/s)	111.75 ± 23.85	120.45 ± 17.01	100.82 ± 28.12	89.61 ± 25.88
VSL (μm/s)	66.13 ± 17.15	94.70 ± 24.83	63.78 ± 20.33	56.84 ± 20.91
STR (%)	59.13 ± 6.72	69.49 ± 5.55	61.14 ± 7.31	61.24 ± 8.44
LIN (%)	49.88 ± 6.69	64.23 ± 7.79	50.00 ± 8.55	49.98 ± 9.90
ALH (μm)	3.58 ± 0.90	2.49 ± 0.57	3.21 ± 0.80	2.84 ± 0.70
BCF (Hz)	3.52 ± 0.79	4.28 ± 0.72	3.71 ± 0.84	3.72 ± 0.94
DSM (sec)	30.43 ± 6.32	32.86 ± 6.33	46.39 ± 9.60	45.98 ± 10.84

Figure S1. Correlation matrix among all the sperm motility traits.

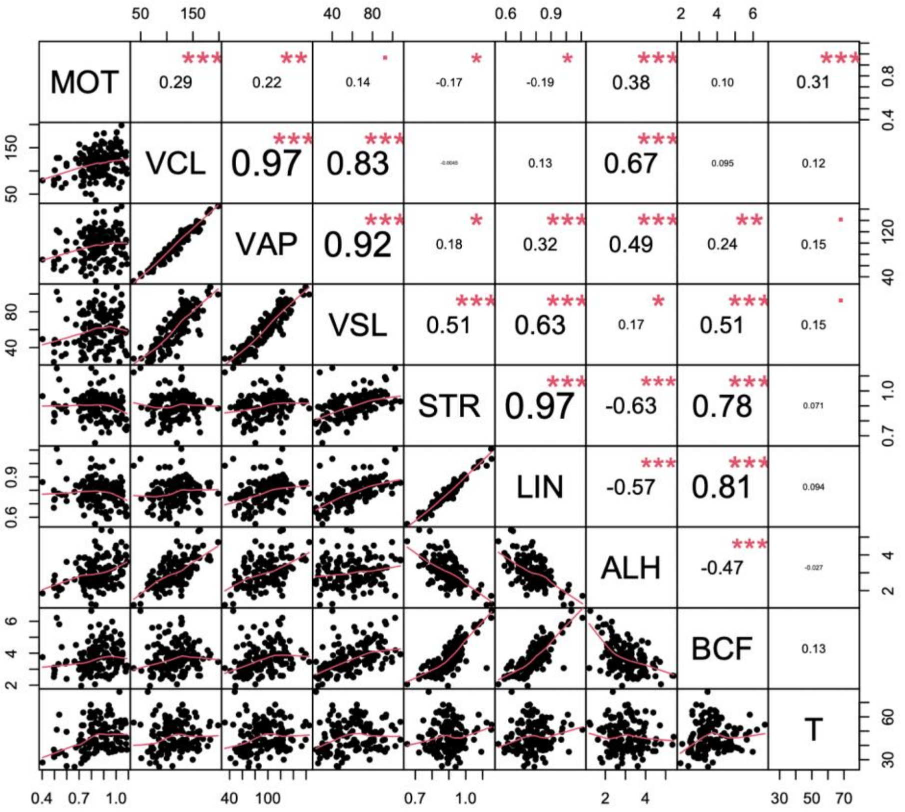

Figure S2. The barplots show the additive male effect on total motility (MOT) and duration (DSM), grouping all treatments.

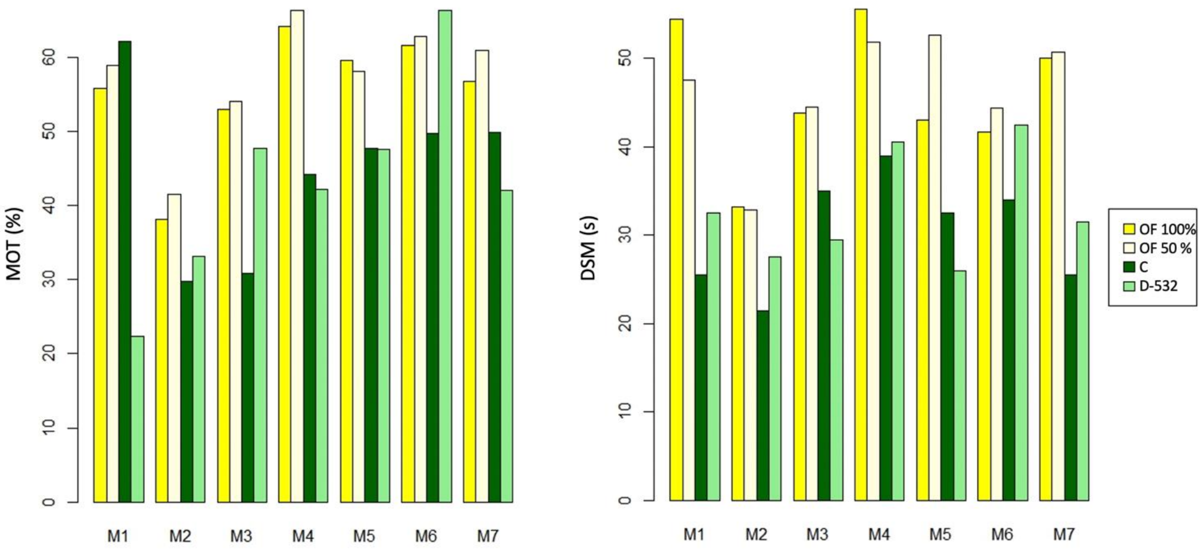

Figure S3. The barplots show the additive female effect (mean of all male crosses) on total motility (MOT) and duration (DSM), grouping OF 50% and OF 100 %. Values registered in C and D-532 are shown.

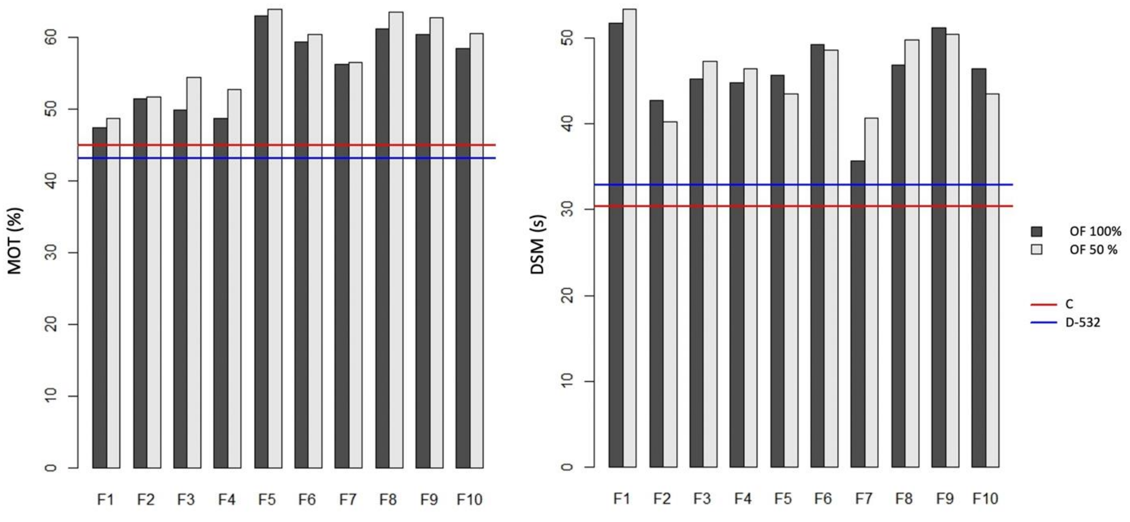

Figure S4. The barplot shows the interaction effect between males and females on total motility (MOT) grouped by male (each colored bar) and female (each group of bars). Both females and males are sorted by their overall means.

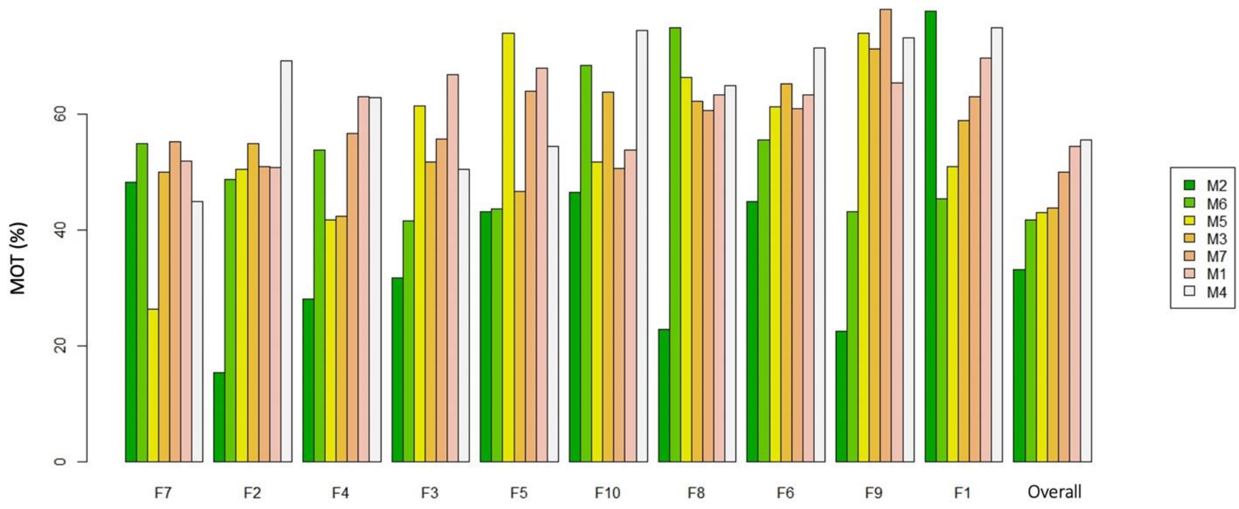
MOT (%)
0
20
40
60
F7
F2
F4
F3
F5
F10
F8
F6
F9
F1
Overall
M2
M6
M5
M3
M7
M1
M4
